# Potentiality of Soybean Proteomics in Untying the Mechanism of Flood and Drought Stress Tolerance

**DOI:** 10.3390/proteomes2010107

**Published:** 2014-03-07

**Authors:** Zahed Hossain, Setsuko Komatsu

**Affiliations:** 1 Plant Stress Biology Lab, Department of Botany, West Bengal State University, Kolkata-700126, India; 2 National Institute of Crop Science, National Agriculture and Food Research Organization, Tsukuba 305-8518, Japan

**Keywords:** soybean, proteomics, drought, flood, review

## Abstract

Dissecting molecular pathways at protein level is essential for comprehensive understanding of plant stress response mechanism. Like other legume crops, soybean, the world’s most widely grown seed legume and an inexpensive source of protein and vegetable oil, is also extremely sensitive to abiotic stressors including flood and drought. Irrespective of the kind and severity of the water stress, soybean exhibits a tight control over the carbon metabolism to meet the cells required energy demand for alleviating stress effects. The present review summarizes the major proteomic findings related to changes in soybean proteomes in response to flood and drought stresses to get a clear insight into the complex mechanisms of stress tolerance. Furthermore, advantages and disadvantages of different protein extraction protocols and challenges and future prospects of soybean proteome study are discussed in detail to comprehend the underlying mechanism of water stress acclimation.

## 1. Introduction

Plants, being sessile organisms, are prone to various environmental stresses. Flooding and drought are the two different forms of water stress that constitute major limiting factors for plant growth, development and quality crop production. Soybean, the world’s most widely grown seed legume, provides an inexpensive source of protein and vegetable oil for human consumption. This important legume crop is adapted to be grown in a wide range of climatic conditions; nevertheless, at seedling stage its growth is significantly affected by several abiotic stressors, including flooding [1,2,3,4,5,6,7,8,9,10,11] and drought [12,13]. 

Dissecting stress tolerance mechanism at molecular level has always been a priority in any crop development program. Stress-induced changes in gene expression modulate metabolic processes through alteration of cellular protein abundance and function. Therefore, understanding how the function of proteins changes under stressed conditions is crucial for clarifying the molecular mechanisms underlying stress tolerance and crop injury. Identification and understanding the biological function of any novel gene conferring such tolerance is a more ambitious goal than merely determining its sequence. Due to lack of correlation between mRNAs’ expression levels and the abundance of their corresponding proteins, proteomic techniques provide one of the best options for the functional analysis of translated regions of the genome. Furthermore, several proteins undergo post-translational modifications such as removal of signal peptides, phosphorylation and glycosylation, that are extremely important for protein function. Hence, a proteomics approach, complemented with genome-sequence data and modern bioinformatics, offers a powerful tool to identify and characterize novel proteins and to follow temporal changes in protein relative abundances under adverse environmental conditions. 

Conventional gel-based proteomic approaches, and gel free-mass spectrometry (MS)-based methods involving label-based and label-free protein quantification have been extensively used for characterization of stress-responsive proteins in soybean [5,6,9,10,11,14,15,16,17]. The present review provides an overview of the major findings related to changes in soybean proteomes in response to flooding and drought stresses to get a clear insight into the complex mechanisms involved in plants stress response. Furthermore, strengths and weaknesses of different proteomic methodologies of extracting complete proteome and challenges and future prospects of soybean proteome study are discussed in detail to comprehend the underlying mechanism of water stress tolerance.

## 2. Protein Extraction

The choice of method for protein extraction largely depends on the type of plant organelle and organs, and/or the nature of desired proteins to be extracted (Table 1). Presence of various interfering substances, such as phenolic compounds, proteolytic and oxidative enzymes, terpenes, organic acids, and carbohydrates create complications during the process of protein extraction, resulting in inferior results such as proteolytic breakdown, streaking, smearing and charge heterogeneity [18]. Elimination of these disturbing compounds during protein extraction is thus necessary to get the optimum result.

**Table 1:** Summary of soybean proteome analyses in response to flood and drought.

Stress	Cultivar/ Stress exposure	Organ/ Organelle	Protein extraction buffer	Protein solubilization /lysis buffer	Proteomic methodologies	Spot resolved Proteins	Differentially abundant protein classification		Ref.
							Function	Localization	
Flooding	Enrei (5 days)	Leaf Hypocotyl Root	10% TCA, 0.07% 2-ME in acetone	8 M urea, 2 M thiourea, 5% CHAPS, 2 mM tributyl-phosphine, 0.4% Ampholytes pH 3–10	IEF, SDS-PAGE, nanoLC-MS/MS	577 (L): 24↑26↓ 555 (H): 35↑31↓ 515 (R): 20↑27↓	Met, Ene, ProtDesSt, DisDef, ProtSyn	Mito, Nucl, Cyto, Extr, ER, Cysk, PM	[9]
	Enrei (2 days)	Hypocotyl Root mitochondria	-	8 M urea, 2% NP-40, 5% 2-ME, 5% PVP 40, 0.4% Ampholytes pH 3–10	IPG, SDS-PAGE, BN-PAGE, nanoLC-MS/MS	Matrix 327 29↑7↓ Membrane 72 5↑11↓	Ene, DisDef	Mito, Chlo	[5]
	Enrei (2 days)	Hypocotyl Root cell wall	-	8 M urea, 2% NP-40, 0.8% Ampholine pH 3.5–10, 5% 2-ME and 5% PVP 40	IEF, SDS-PAGE, MALDI-TOF MS, nanoLC-MS/MS, protein sequencing	204 4↑12↓	Met, ProtDesSt, DisDef	Sec	[7]
	Enrei (1–4 days)	Hypocotyl Root	Phosphate saline buffer pH 7.6, 400 mM NaCl, 3 mM NaN_3_ followed by 10% TCA	8 M urea, 2% NP-40, 0.8% Ampholine (pH 3.5–10), 5% 2-ME and 5% PVP 40	IEF/IPG, SDS-PAGE, MALDI-TOF MS, protein sequencing	803 21↑7↓	ProtDesSt, DisDef, Ene, Pmet, CellSt, Trans	-	[8]
	Asoagari (3, 7 days)	Root	Cold acetone containing 10% TCA, 0.07% 2-ME	8 M urea, 1% CHAPS, 0.5% IPG buffer pH 4–7, 20 mM DTT, BPB	IPG, SDS-PAGE, MALDI-TOF MS, ESI-MS/MS	~900 14↑5↓5 Newly induced	Met, Ene, DisDef, ProtSyn	-	[19]
	Enrei (12–48 h)	Hypocotyl Root	-	9.5M urea, 2% NP-40, 2% Ampholines pH 3–10, 5% 2-ME	IEF/IPG tube gel, 2-DE, MALDI-TOF MS, nanoLC-MS/MS, protein sequencing	799 14↑20↓	Ene, DisDef, Pmet, CellSt, Secmet, Sgnl	-	[20]
	Enrei (1 days)	Hypocotyl Root plasma membrane	-	8 M urea, 2% NP-40, 0.8% Ampholine pH 3.5–10, 5% 2-ME and 5% PVP 40	IEF tube gel, 2-DE, MALDI-TOF MS, nanoLC-MS/MS, protein sequencing	150 12↑2↓	ProtDesSt, ProtSyn, DisDef, CellDiv, Trans, Pmet, Ene, Secmet, Sgnl	-	[21]
Flooding Low oxygen	Enrei (3, 6 days Low oxygen)	Root	10% TCA, 0.07% 2-ME in acetone	8 M urea, 2 M thiourea, 5% CHAPS, 2 mM tributyl-phosphine, 0.4% Ampholytes pH 3–10	IEF, SDS-PAGE , MALDI-TOF MS, nanoLC-MS/MS	1,233 F: 4↑12↓ LO: 2↓	Met, Ene, ProtDesSt, Sgnl, ProtSyn, DisDef	Cyto, Chlo, Nucl	[10]
Drought	Enrei (Stop watering 10% PEG 4 days)	Leaf Hypocotyl Root	10% TCA, 0.07% 2-ME in acetone	8 M urea, 2 M thiourea, 5% CHAPS, and 2 mM tributyl-phosphine, 0.4% Ampholytes pH 3–10	IPG, SDS-PAGE, nanoLC-MS/MS	549 (L): PEG: 20↑17↓ Drought: 20↑21↓ 451 (H): PEG: 20↑13↓ Drought: 18↑19↓ 632 (R): PEG: 20↑10↓ Drought: 33↑16↓	Met, Ene, ProtSyn, DisDef	Chlo, Cyto, Nucl, Mito	[12]
	Taegwang (withholding water - 5 days, rewatering - 4 days)	Root	Mg/NP-40 buffer [0.5 M Tris-HCl ( pH 8.3), 2% NP-40, 20 mM MgCl2, 1 mM PMSF , 2% 2-ME, 1% PVP], water-saturated phenol, followed by ammonium acetate in methanol	8 M urea, 1% CHAPS, 0.5% IPG buffer (pH 4–7), 20 mM DTT, BPB	IPG, SDS-PAGE, MALDI-TOF MS	1,350 6↑20↓2 New	Met, Ene, Sgnl, DisDef, CellSt,	-	[13]
Osmotic stress	Enrei (10% PEG 1–4 days)	Hypocotyl Root plasma membrane	Plasma membrane proteins precipitated by TCA followed by cold acetone washing	7 M urea, 0.2 M thiourea, 0.2mM tributylphosphine, 5% PVP-40, 0.4% CHAPS, 0.2% Ampholytes (pH 3.0–10.0)	IEF tube gel, SDS-PAGE, LC MS/MS, nanoLC-MS/MS	202 11↑75↓	Sgnl, Met, ProtSyn, DisDef, Trans	-	[22]
Osmotic stress	Enrei (0, 5, 10, 20% PEG 1–4 days)	Root	Phosphate saline buffer (pH 7.6): 65 mM K_2_HPO_4_, 2.6 mM KH_2_PO_4_, 400 mM NaCl and 3 mM NaN_3_ followed by 10% TCA	8 M urea, 2% NP-40, 0.8% Ampholine (pH 3.5–10), 5% 2-ME and 5% PVP 40	IEF tube gel, SDS-PAGE, MALDI-TOF MS, protein sequencing	415 19↑18↓	DisDef, Ene, ProtDesSt, Met, CellSt, Secmet.	-	[23]

Up and down arrows indicate stress-induced increase and decrease in protein abundance respectively. Abbreviations: BPB, bromophenol blue; CBB, Coomassie brilliant blue; DTT, dithiothreitol; IPG, immobilized pH gradient; IEF, isoelectric focusing; LC, liquid chromatography; MS, mass spectrometry; PMSF, phenyl methyl sulfonyl fluoride; PVP, Polyvinylpyrrolidone; TCA, trichloroacetic acid; 2-ME, 2-mercaptoethanol. L, H and R represent leaf, hypocotyl and root respectively. Functional classification: Met, metabolism; Ene, energy; ProtDesSt, protein destination/storage; ProtSyn, protein synthesis; DisDef, disease/defense; CellDiv, cell division; Trans, transporter; Pmet, primary metabolism; Secmet, secondary metabolism; CellSt, cell structure; Sgnl, signal transduction; GrDev, growth and development; TrStr, translocation and storage. Subcellular localization: Chlo, chloroplast; Mito, mitochondria; PM, plasma membrane; Nucl, nuclear; Cyto, cytoplasm; Extr, extracellular matrix; ER, endoplasmic reticulum; Cysk, cytoskeleton; Vacu, vacuolar; Sec, secretory pathway.

Soybean seeds contain a large amount of secondary metabolites like kaempferol and quercetin which not only hampers high-quality protein extraction, but also impedes protein spot separation in high resolution two-dimensional polyacrylamide gel electrophoresis (2-DE) gels, resulting in a significant reduction in the number of distinctly resolved protein spots [24,25]. Furthermore, presence of abundant storage proteins such as β-conglycinin and glycinin often hinders the isolation and characterization of less abundant seed proteins. Sample fractionation technique has proved to be an efficient strategy for successful removal of such highly abundant storage proteins. With the simple addition of 10 mM calcium chloride to the salt soluble soybean seed protein extract in low ionic strength buffer, the a, a', and β subunits of β-conglycinin and the acidic and basic subunits of glycinin were found to be reduced significantly from the total protein extract [26]. For extracting soybean seed proteins both at mature [27] and seed filling stages [28], phenol based protein extraction method was reported to be more effective. As compared to the trichloroacetic acid (TCA)/acetone or Tris–HCl buffer, protein extracted in buffer comprises of 50% phenol, 0.45 M sucrose, 5 mM EDTA, 0.2% 2-mercaptoethanol, 50 mM Tris-HCl (pH 8.8) produced a large number of reproducible protein spots. Natarajan *et al*. [29] also compared four different protein extraction/solubilization methods-urea, thiourea/urea, phenol, and a modified TCA/acetone to determine their effectiveness in separating soybean seed proteins by 2-DE. The thiourea/urea and TCA methods were found to be more suitable in resolving less abundant and high molecular weight proteins. In addition, these two methods exhibited higher protein resolution and spot intensity as compared to the rest of the methods. Recently, Barbosa *et al*. [30] successfully analysed mature seed proteome by extracting proteins in 50 mM Tris-HCl (pH 8.8), 1.5 mM KCl, 10 mM dithiothreitol (DTT), 1.0 mM phenylmethylsulfonyl fluoride (PMSF), and 0.1% SDS followed by precipitation in 0.1 M ammonium acetate in methanol. 

To compare soybean leaf and flower proteomes at different developmental stages, Ahsan and Komatsu [31] evaluated three different protein extraction protocols-TCA precipitation [32], phenol extraction method [33] with modifications and direct tissue homogenizing in suitable protein solubilization buffers. To optimize protein pellet solubilization buffer, A-buffer containing 8 M urea, 2% Nonidet P-40, 2% ampholine (pH 3.5–10), 5% 2-mercaptoethanol, and 5% polyvinylpyrrolidone (PVP)-40; B-buffer [32] containing 7 M urea, 0.2 M thiourea, 0.2 mM tributylphosphine (TBP), 0.4% CHAPS, 5% PVP-40, and 2% ampholine (pH 3–10); and C-buffer containing 8.5 M urea, 2.5 M thiourea, 5% CHAPS, 1% DTT, 1% Triton X-100, and 0.5% ampholines (pH 3–10 and 5–8) were tested. A combination of the phenol-based method with C-solubilization buffer generated high quality proteome maps in terms of well-separated resolved spots, spot intensity, and the number of proteins in the 2-DE gels with no horizontal streaking and high background noise levels. 

For root proteomic analysis, TCA/acetone precipitation is the most widely used protein extraction method. Root proteins extracted in 10% TCA and 0.07% 2-mercaptoethanol in acetone followed by subsequent solubilization in the lysis buffer containing 8 M urea, 2 M thiourea, 5% CHAPS, and 2 mM TBP results in a high quality gel with a good number of resolved protein spots [16,34]. Addition of DTT and PVP in the soybean protein extraction buffer was found to be effective in enhancing the number of resolved spots in gels [35,36]. Ahsan and Komatsu [31] reported that treatment of root with Mg/Nonidet P-40 buffer followed by extraction with alkaline phenol and methanol/ammonium acetate produced high-quality proteome maps consisting of numerous well-separated spots with high intensity, on 2-DE gels.

On the whole, instead of having physicochemical limitations of each and every protocol, the TCA/acetone precipitation and phenol-based protocols are the most reliable and efficient protein extraction methods for various soybean organs to obtain high quality gels [37,38] (Table 1).

## 3. Changes in Soybean Proteome in Response to Flooding

Soil oxygen deprivation, the most inevitable consequence of flooding, forces submerged plants to shift from aerobic to anaerobic respiration [39,40]. This metabolic swing helps plants to regenerate NAD^+^ through ethanol fermentation by selectively synthesizing flooding-inducible proteins involved in sucrose breakdown, glycolysis, and fermentation [41]. The suppressed energy metabolisms accelerate energy depletion resulting in growth retardation, and render flooded plants vulnerable to other biotic and abiotic stresses.

**Figure 1:**
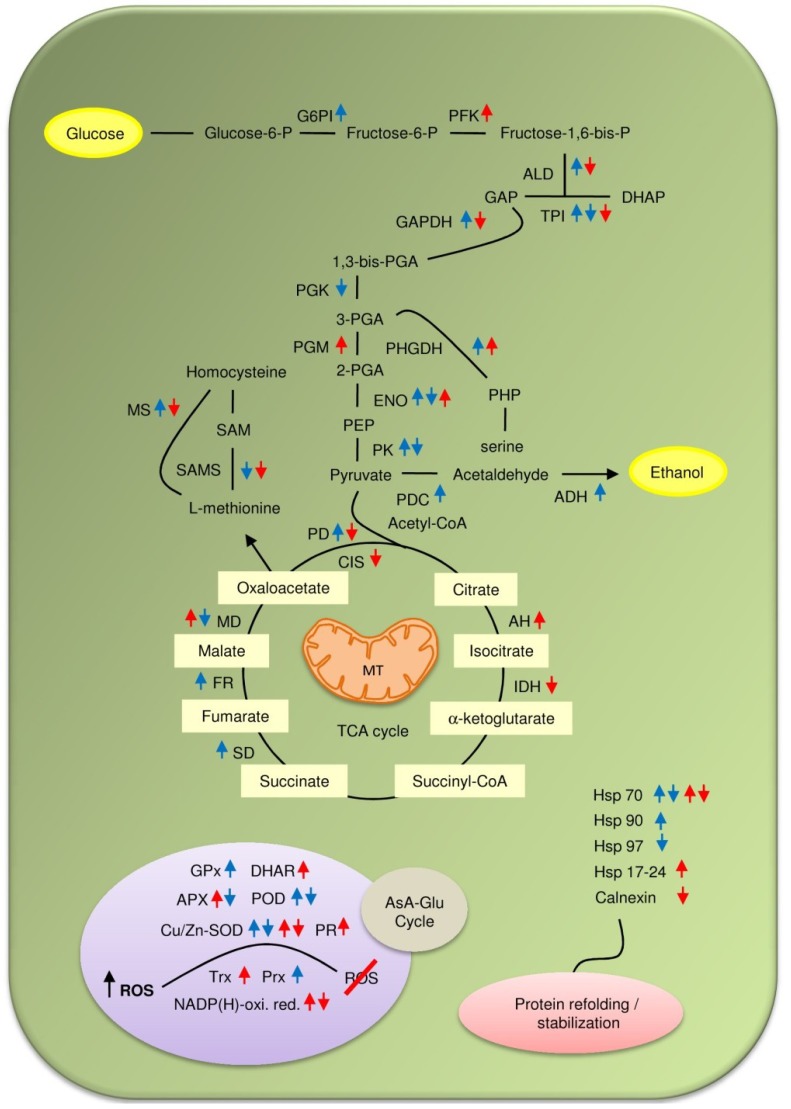
Water stress mediated changes in metabolic pathways. Blue and red arrows indicate changes in protein abundance (upward arrows indicate increase and downward arrows indicate decrease) in response to flooding and drought, respectively.

*Abbreviations:* ADH; alcohol dehydrogenase; AH, aconitate hydratase; ALD, aldolase; APX, ascorbate peroxidase; DHAR, dehydroascorbate reductase; ENO, enolase; FR, fumarase; GAPDH, glyceraldehyde 3-phosphate dehydrogenase; G6PI, Glucose-6-phosphate isomerase; GPx, glutathione peroxidase; GR, glutathione reductase; Hsp, heat shock proteins; IDH, isocitrate dehydrogenase; MD, malate dehydrogenase; PD, pyruvate dehydrogenase; PDC, pyruvate decarboxylase; PFK, Phosphofructokinase; PGK, phosphoglycerate kinase; PGM, phosphoglycerate mutase; PHGDH, 3-Phosphoglycerate dehydrogenase; PHP, 3-phosphohydroxypyruvate; PK, pyruvate kinase; PR, pathogenesis-related; POD, peroxidase; Prx, Peroxidoxin; ROS, reactive oxygen species; SAMS, S-adenosylmethionine synthetase; SAM, S-adenosylmethionine; SD, succinate dehydrogenase; TPI, triose-phosphate isomerase; Trx, thioredoxin.

Different physiological and molecular aspects of plant response toward flooding stress are well documented. In this section, contribution of proteomic studies to flooding stress mediated modulation of protein networks have been summarized for better understanding of flood sensing and tolerance mechanism both at organ and whole plant level (Figure 1). 

Organ-specific proteome response of soybean seedlings under flooding stress has been well analyzed [1,2,5,6,7,8,9,10,16,42] (Table 1). Root represents the first organ of a plant in sensing waterlogged condition. Thus, root has always been a target of proteomic investigation to elucidate the plants’ flood response mechanism. Root proteome study of submerged young soybean seedlings revealed that glycolysis related proteins including UDP-glucose pyrophosphorylase and fructose-bisphosphate aldolase, disease/defense-related proteins such as ROS (reactive oxygen species) scavengers, chaperones, hemoglobin, and/or acid phosphatase were mostly affected [16,20,42]. A separate study by Alam *et al*. [19] has shown higher expression of glycolysis and fermentation pathways related proteins in roots of three-week-old seedlings. Analysis of enzyme activities and carbohydrate contents in flooded seedlings further confirmed that glucose degradation and sucrose accumulation accelerated during flooding due to activation of glycolysis and decrease of sucrose degrading enzymes [16]. In addition, the methylglyoxal pathway, the detoxification route linked to glycolysis, was found to be increased under flooding.

Flood-specific accumulation of alcohol dehydrogenase (ADH2) in roots of soybean indicates activation of alcohol fermentation pathway to cope with the hypoxic condition [6]. A recent proteomic study on flooding-tolerant mutant line showing better root growth under flooded condition revealed higher abundances of fermentation-related proteins including different types of ADHs and pyruvate decarboxylase isozymes on exposure to submergence [1]. Additionally, no changes in the cell wall loosening-related proteins were observed under flooding stress, thereby preserving the viability of the root tip and permitting rapid growth at post-stress period. 

Within the root system, the tip portion of the primary roots plays an essential role in seedling establishment. Flooding induced cell death in the root tip region and a subsequent suppression in root elongation have been reported in flooded soybean seedlings [17]. Predominant proteins involved in stress response, glycolysis, redox homeostasis, and protein processing found to be located in differentiated root zones including root apex with different abundances [36]. Gel free MS based quantitative proteomics and phosphoproteomics approaches have been well exploited to enumerate the altered protein relative abundance profiles of soybean root tips under flooding stress [17]. Classification of differentially accumulated proteins revealed that majority of the proteins involved in glycolysis, fermentation, cell wall metabolism and nucleotide metabolism were increased; while, the relative abundance of most of the proteins involved in amino acid metabolism and cell organization were decreased. In addition, few proteins including sucrose-binding protein, phosphatidylinositol-4-phosphate 5-kinases, actins, and alpha-tubulins, were found to be accumulated specifically in the root tip region. Accumulation of sucrose-binding proteins in flooded soybean root tips suggests an enhanced sucrose accumulation. This observation is in agreement with the finding, reported previously in soybean roots and hypocotyls [16]. Furthermore, Yanagawa and Komatsu [43] reported that flooding, and not the hypoxic condition, was responsible for the root tip degradation resulting from ubiquitin/proteasome-mediated proteolysis, as these injuries were independent of the oxygen concentration. It is believed that the Ub/proteasome-mediated proteolysis of enzymes involved in glycolysis and fermentation pathways may be negatively controlled under the hypoxic condition caused by flooding [3]. Previous gel and gel-free MS based proteomic study by Nanjo *et al*. [16] has also revealed differential regulation of 20S proteasome subunits in flooded soybean. Altered expression of each 20S proteasome subunit in response to flooding stress may thus affect the amount as well as the activity of the 26S proteasome, thereby altering flooding tolerance. 

Among the differentially expressed ROS scavenger proteins, cytosolic ascorbic peroxidase (cAPX) and superoxide dismutase (SOD) were found to be decreased in response to flooding. Proteomic screening of six different soybean cultivars revealed a significant decrease in cAPX 2 proteins on exposure to flooding [44]. Abundance of cAPX 2 transcripts was also found to be decreased significantly after flooding, as did the APX activity. Results suggest that cytosolic APX 2 plays a key role in flood-induced stress response of young soybean seedlings.

The post-stress recovery period is equally critical phase for the ultimate survival of a stressed plant. Salavati *et al*. [34] examined the proteome change under post-flooding recovery stage in soybean roots. Clustering analysis based on the expression profiles of the differentially abundant protein spots revealed that flooding resulted in a decrease of ion transport-related proteins and an increase of proteins involved in cytoskeletal reorganization, cell expansion, and programmed cell death. The observed changes in protein relative abundance suggest that the regulation of root growth through cell wall modification and the synthesis of S-adenosylmethionine-related metabolites may be involved in post-flooding recovery processes in soybean seedlings. 

Flood-induced reduction in plant biomass is directly related to stomatal limitations on net photosynthesis that result in reduced carbon assimilation [45]. Restriction in photosynthetic activity is also influenced by changes in the photosynthetic components, such as ribulose-1,5-bisphosphate carboxylase/oxygenase (RuBisCO) and other photosynthesis-related proteins [46]. Leaf proteome analysis of soybean seedlings revealed that most of decreased proteins were involved in energy production and primary/secondary metabolism [47]. This observation is in agreement with the results of a recent gel-based organ-specific proteomic study by Khatoon *et al*. [9]. As compared to the roots and hypocotyls, more metabolism, energy and disease/defense related proteins were found to be decreased in leaves. The reduced levels of isoflavone reductase and other disease/defense-related proteins (SOD, CAT) in the roots and leaves of flooded seedlings compared to non-stressed seedlings indicate that the defense response is highly suppressed in soybean seedlings under flooding stress. Furthermore, a decreased relative abundance of chlorophyll *a*-*b* binding proteins were recorded. Overall, reduced photosynthetic activity along with low expression of ROS scavenging proteins lead to suppression of seedling growth under flooding.

As compared to whole organ proteome study, an in-depth investigation of subcellular organelles proteomes generates much detailed information about the intrinsic mechanism of stress response as it correlates the possible relationship between the protein abundance and plant stress tolerance. The intracellular organelles and compartments and their interactions during the stressed condition represent the primary defense response. Among the organelles, mitochondria have been a target for subcellular proteomic study, as most of the abiotic stresses primarily impair mitochondrial electron transport chain resulting in excess ROS generation. Proteomic technique coupled with metabolomics has been successfully used to study the flooding stress effects on mitochondrial function of flooded soybeans [5]. Flooding stress caused a considerable impairment of the electron transport chain in the roots and hypocotyls of soybean seedlings. Abundance of inner membrane carrier proteins and proteins related to complexes III, IV, and V of the electron transport chain were found to be decreased, while proteins and metabolites related to TCA and γ-amino butyrate (GABA) shunt were increased under flooding stress resulting in high NADH production. In addition, succinate-semialdehyde dehydrogenase and GABA were significantly increased by flooding stress, as was 2-oxoglutarate dehydrogenase, suggesting that the GABA shunt is involved in a replenishment of intermediates needed for energy production that have been depleted by flooding stress. 

Plant cell wall plays an essential role in stress sensing and signal transduction between the apoplast and symplast. Investigation on the function of the cell wall of flooded soybean seedlings revealed decrease in lipoxygenases, germin-like protein precursors, stem glycoprotein precursors and Cu–Zn SOD [7]. Proteome analysis suggested that flooding caused suppression of lignifications in roots through a decrease of ROS scavenging enzymes and jasmonate biosynthesis. Similarly, alterations in the plasma membrane proteins of soybean exposed to flooding stress were analyzed using gel-based and gel-free proteomics techniques [21]. Plasma membrane acts as a primary interface between the cellular cytoplasm and the extracellular environment and thus plays a vital role in cell communication. Among the stress induced novel proteins, SOD was found to be remarkably increased, suggesting that the antioxidative system may play a crucial role in protecting cells from oxidative damage following exposure to flooding stress. In addition, flood induced an enhanced accumulation of heat shock cognate 70 kDa protein which might protect proteins from denaturation and degradation during flooding stress.

In a recent gel-free proteomic study by Komatsu *et al*. [2], exogenous application of phytohormone abscisic acid (ABA) at early seedling stage has been found to be effective in enhancing flood tolerance in soybean. The abundance of 34 nuclear proteins such as histone deacetylase and U2 small nuclear ribonucleoprotein increased by ABA supplementation under flooding; while, 35 nuclear proteins such as importin alpha, chromatin remodeling factor, zinc finger protein, transducin, and cell division 5 protein were decreased. In addition, mRNA expression levels of cell division cycle 5 protein, C_2_H_2_ zinc finger protein SERRATE, CCCH type zinc finger family protein, and transducin were found to be down-regulated under the ABA treatment. Authors suggested that ABA might be involved in the enhancement of flooding tolerance through the control of energy conservation via glycolytic system and the regulation by zinc finger proteins, cell division cycle 5 protein and transducin. Similar nuclear proteomic analysis by Oh *et al*. [48] reported acceleration of protein poly-ADP-ribosylation and suppression of RNA metabolism in root tips of young soybean seedlings under flooding stress. A separate proteomic study on endoplasmic reticulum (ER)-enriched fraction of flooded soybean root tips revealed decreased abundances of proteins involved in stress, hormone metabolism, cell wall and DNA repairing [4]. Additionally, expression of luminal-binding protein 5 was specifically induced under flood stress, while arabinogalactan protein 2 and methyltransferase PMT2 were found to be down-regulated. Overall, results indicate that flooding predominantly affects protein synthesis and glycosylation in the ER of soybean root tips. 

Taken together, these results suggest that the tight metabolic regulation over the energy consumption and quick activation of plant defense system are essential to conquer the flooding stress.

## 4. Drought Induced Modulation of Soybean Proteome Composition

Drought constitutes another form of water stress that results from scarcity of water around the root zone. Like other legumes, soybean is also sensitive to drought condition. Decline in photosynthetic carbon gain as a result of stomatal closure or due to a decrease in RuBisCO activity is one of the major reasons behind the loss of crop productivity during drought phase [49]. The activity of the photosynthetic electron transport chain is finely tuned to the availability of CO_2_, and photosystem II activities often decline in parallel under drought conditions [50]. In soybean, photosynthesis decreases by about 70% during severe water stress, although the respiration rate is not that much affected [51]. Drought tolerance has always been considered as one of the top priorities for soybean improvement [52]. The genetic complexity of drought tolerance, the lack of efficient selection technique, environmental variability, and the strong interactions between genotype and water availability are some of the key limiting factors for designing drought tolerant soybean cultivars [13].

Different aspects of plant response toward dehydration stress have been well documented. However, information on drought sensing and tolerance mechanism at the proteome level is very limited. In this section, published proteomic works on dehydration stress mediated changes in soybean proteomes are summarized for better understanding of the drought stress responsive mechanism (Figure 1). The functional categorization revealed that most of the drought-responsive proteins were chiefly involved in redox regulation, oxidative stress response, signal transduction, protein folding, secondary metabolism, and photosynthesis. 

Root is found to be the most drought-responsive organ showing maximum changes in protein abundance in response to stress. Polyethylene glycol (PEG), a high molecular weight osmotic substance, is frequently used to simulate drought stress in soybean lowering the water potential in a similar way to soil drying [22,23]. Changes in relative abundance of metabolism-related proteins were shown to be increased in leaves of both PEG-treated and drought-stressed seedlings, while proteins related to energy production- and protein synthesis were decreased [12]. In a separate study, abundance of proteins associated with a wide variety of cellular functions, including carbohydrate and nitrogen metabolism, cell wall modification, signal transduction, cell defense and programmed cell death were found to be highly affected in soybean roots subjected to severe but recoverable drought stress at seedling stage [13]. 

Toorchi *et al*. [23] studied the PEG-induced osmotic stress related proteins in soybean roots using a 2-DE gel based proteomic approach. Osmotic stress is just a simulation of drought condition, where high concentration of osmolyte e.g. PEG stimulates the roots to look for unexplored water. This results in continuing root growth and a delay in root lignification. Protein identification revealed a decrease in caffeoyl-CoA 3-*O*-methyltransferase, Hsp-70, S-adenosylmethionine synthetase with high abundance of disease/defense associated proteins [23]. In plant cell wall, lignin is the major structural component of secondary thickening that imparts mechanical strength to stems and roots, and hydrophobicity to water-conducting vascular elements. Caffeoyl-CoA-O-methyltransferase is involved in the lignification process and its decreased abundance in soybean roots under osmotic stress thus might result in the reduction of lignin content as an adaptive response to osmotic stress. In separate proteomic investigation, comparative analysis of plasma membrane proteins of two-day-old soybeans under PEG-mediated osmotic stress revealed an increase in transporter proteins, indicating a high rate of ion efflux by the plasma membrane bound H^+^-ATPase [22].

In addition, calnexin protein was found to be highly increased under stress. Nevertheless, decreased expression of calnexin was reported in 14-day-old soybean roots under 10% PEG treatment [53]. Calnexin is an ER-localized molecular chaperone protein, involved in folding and quality control of proteins. This protein interacts with many nascent membrane and soluble proteins of the secretory pathway and participates in the folding and quality control of newly synthesized glycoproteins [54]. Authors suggested that calnexin interacts with a 70 kDa heat shock cognate protein and probably functions as a molecular chaperone under PEG-induced osmotic stress.

Overall, drought or PEG-mediated osmotic stress at seedling stage affects a wide range of cellular functions, including carbohydrate and nitrogen metabolism, cell wall modification, signal transduction, cell defense and programmed cell death in soybean. Proteomic findings of drought stressed soybean indicate that proteins associated with osmotic adjustment, defense signaling and programmed cell death play key roles in drought adaptation.

## 5. Novel Methodological Approaches to Study Plant Proteomes

The ultimate success of any proteomic approach depends upon various factors including isolation of full component of proteins, separation, visualization and their accurate identification. In spite of recent advancement, more emphasis needs to be given on the protein extraction protocols, in particular for very low and high abundance proteins. In soybean root, identification of low abundance of signaling proteins, transcription factors and their protein complexes is often a challenge for 2-DE based proteomic techniques. Nanjo *et al*. [17] adapted a gel free analysis of complete root-tip proteome, in which protein samples were reduced and alkylated in a denaturing solution followed by trypsin digestion. Trypsin-digested samples were then injected on nanoLC coupled to MS/MS. This method allows detection of MS peaks with up to 5000 times differences in abundance. In order to determine the composition of plant protein complexes, Smaczniak *et al*. [55] used another, rather more sensitive fluorophore-tagged protein immunoprecipitation and label-free MS-based quantification techniques to facilitate identification of low abundance signaling and regulatory protein complexes from native plant tissues. Furthermore, an advanced technique like laser-capture micro-dissection [56] for tissue proteomics could be used further for accurate identification of tissue- and cell-specific proteins involved in plant response to abiotic stresses. Gil-Quintana *et al*. [57] reported proteomic analysis of root nodules of drought stressed soybean using shotgun proteomics technique. In order to have complete proteome of root nodules, including all low abundance proteins, protein digests were analysed via shotgun nano-LC-ultra using a monolithic reversed-phase column directly coupled to an Orbitrap XL mass spectrometer [58].

Similarly, in leaf, presence of extremely abundant photosynthetic CO_2_ fixation enzyme RuBisCO not only limits the dynamic resolution and yield of low abundance proteins of interest but also masks other proteins or affects the electrophoretic migration of neighboring protein species [59]. Different fractionation techniques based upon different physiological or biochemical principles have been proposed to deplete or reduce a substantial portion of RuBisCO from total leaf protein extract [60,61]. Ahsan *et al*. [62] used a PEG-fractionation method to eliminate RuBisCO during protein extraction from tomato leaves. In this method, proteins were first extracted using Mg/Nonidet P-40 buffer consisting of 0.5 M Tris-HCl, 2% Nonidet P-40, 20 mM MgCl_2_, 2% 2-mercaptoethanol, 1 mM PMSF, and 1% PVP, and were then fractionated with 15% PEG. Furthermore, anti-RuBisCO LSU antibody affinity column with protein A-Sepharose as a resin has been successfully used for effective elimination of RuBisCO [63]. In comparison of these complex and lengthy methods, Krishnan and Natarajan [64] developed a fast and simple fractionation technique using 10 mM Ca^2+^ and 10 mM phytate to precipitate 85% of the RuBisCO from soybean leaf soluble protein extract. Recently, Khan *et al*. [65] also reported a modified protein extraction method for effective removal of RuBisCO. In this method, leaves were homogenized in buffer mixture containing 50% extraction buffer (100 mM Tris-HCl (pH 8.0), 100 mM EDTA, 50 mM borax, 50 mM vitamin C, 1% PVP-40, 1% triton X-100, 2% 2-mercaptoethanol and 30% sucrose) and 50% solubilization buffer (8.5 M urea, 2.5 M thiourea, 5% CHAPS, 1% DTT), 1% triton X-100 and 0.5% ampholin (pH 3–10 and 5–8)) followed by incubation on ice for 1 h and precipitation by adding 20% TCA. The 2-DE pattern revealed displaced RuBisCO LSU to a new position with low molecular weight and pI value. 

As compared to whole organ, in-depth sub-cellular organelle proteome study generates much detailed information on the intrinsic mechanism of plants’ abiotic stress responses. One of the most challenging aspects of subcellular proteomics is the proper isolation of the concerned organelle from the total tissue extract. The conventional methods of subcellular fractionation typically involve differential and density-gradient centrifugation, using a series of centrifugation steps to separate different populations of cellular compartments or organelles from cell homogenates based on their mass and/or density. Nevertheless, the resolving power of differential centrifugation is comparatively poor and may result in fractions containing different organelles having similar sedimentation velocities [66]. In contrast, density-gradient centrifugation has been extensively used in organellar proteomics studies. This method separates organelles based on continuous or discontinuous gradients using various media, such as sucrose, Ficoll, Percoll, Nycodenz and Metrizamide of different osmolarities, viscosities or densities. 

Free-flow electrophoresis (FFE) is another alternative strategy for fractionation of organelles based on their net global isoelectric charges or electrophoretic mobilities. Immunoaffinity purification is a more advanced technique to isolate organelles with specificity and in adequate yields [67]. Both affinity purification and immunoprecipitation methods are based on principle of binding immobilized ligands (such as antibodies) with that of targets (organelle of interest). Fluorescent-assisted organelle sorting (FAOS) is the most emerging sophisticated organelle isolation technique that works on the principle of flow cytometry. This organelle specific marker protein based approach has been found to be effective in mitochondria [68] and vesicles [69] sorting for proteomic analysis. Similarly, subtractive proteomics approach is capable of precise assigning and identifying proteins to their specific subcellular locations. This method effectively eliminates target organelles contamination from co-purifying organelles. It compares and subtracts the identified protein constituents of the contaminated fraction containing the organelle of interest against that of a crude preparation [70].

Protein phosphorylation is the best-studied posttranslational modification that plays a pivotal role in signal transduction cascade. Identification of kinases, their substrates, and the specific site of phosphorylation is thus a key to molecular understanding of stress signaling. The MS-based phosphoproteomic technology has become an invaluable tool for the identification of phosphoproteins and mapping of phosphorylation sites. Nevertheless, identification of *in vivo* phosphorylation sites of individual proteins of interest, necessity for their functional characterization is a big challenge for any phosphoproteomic study. Phosphorylated proteins represent only a small fraction of the whole proteome, thus demanding an effective enrichment method prior to quantification and identification [71]. Immobilized metal affinity chromatography (IMAC) and immunoprecipitation using antibodies against phosphorylated amino acids are the two well known pre- fractionation techniques largely employed before MS analysis. Much progress has been made in quantitative and dynamic analysis of mapped phosphorylation sites in recent time. This method comprised of the isolation of phosphopeptides by IMAC followed by MS/MS or MS(n) analysis has enabled detection of hundreds of *in vivo* phosphorylation sites [72]. Phosphopeptides have been successfully isolated from complex mixtures with strong cationic exchange (SCX) chromatography [73] or strong anionic exchange (SAX) chromatography followed by IMAC [74]. Phosphoproteomics analysis of the Arabidopsis plasma membrane led to the identification and characterization of more than 300 phosphorylation sites [75]. The majority of phosphorylation sites of the membrane transporters have been found to be conserved among putative orthologs and to a lesser extent among some members of the same protein family. On the other hand, affinity purification of phosphoproteins with phospho-specific antibodies such as anti-phosphoserine/threonine prior to MS has limited applications in plants. Recent development in the specific labelling techniques greatly helps in the quantification of phosphorylation profiles and their stress-induced changes with the passage of treatment time. The iTRAQ and SILAC labelling have been found to be most successful in combination with IMAC and MS [72]. These techniques label peptides at the final stage before MS *in vitro* or label proteins during cell growth *in vivo*, respectively, and enable the measurement of changes of individual phosphorylation sites during a time-course stress experiment. Hsu *et al*. [76] compared both label-free LC-MS and stable isotope labelling LC-MS methods for quantitative analysis of phosphorylation sites in membrane fractions of salt stressed Arabidopsis. The functional phosphoproteomic analysis led to a successful identification of novel salt stress-responsive protein phosphorylation sites from membrane isolates of salt-stressed plants by membrane shaving followed by Zirconium ion-charged magnetic beads, and tandem MS analyses. 

Moreover, introduction of Pro-Q Diamond dye based fluorescence-linked assay has opened new avenues in a large-scale quantitative analysis of phosphoproteins. Pro-Q Diamond has been successfully used to specifically label and detect phosphoserine-, phosphothreonine-, and phosphotyrosine-containing proteins directly in SDS-polyacrylamide gels and 2-DE gels. Nanjo *et al*. [17] successfully exploited Pro-Q Diamond phosphoprotein dye technology in determining flooding induced changes in phosphorylation status of proteins involved in energy generation, protein synthesis and cell structure maintenance in root tips of soybean seedlings. 

Compared to phosphoproteomics, plant redox proteomics study of oxidatively modified proteins is more challenging, due to technical limitations such as maintaining the *in vivo* redox states of proteins and the lability of certain PTMs during sample preparation and mass spectrometric analysis [77]. To balance redox metabolism, cells possess a redox signaling network that can sense environmentally induced redox imbalances and initiates compensatory responses either to readjust redox homeostasis or to repair oxidative damage [78]. Within plant cell, chloroplasts, mitochondria and peroxisomes are the primary sites of ROS/ RNS (reactive nitrogen species) generation and the NADPH oxidase located at the plasmalemma, and the cell wall/apoplast peroxidases, amine oxidases, and oxalate oxidases are important components of the ROS-generating system. The highly dynamic and robust ROS gene network that encodes both ROS-producing and ROS-scavenging proteins plays an essential role in monitoring and controlling cellular ROS levels in addition to ROS mediated signalling [79]. Oxidative or nitrosative stress leads to redox modifications of proteins, and may be reversible such as oxidation of cysteines to disulphides or sulphenic acids or irreversible modifications, e.g., carbonylation, oxidation of cysteines to sulphonic acids, oxidation of tryptophan [80]. 

Among the available high*-*throughput techniques, redox proteomics has been found to be the best-suited approach for identifying and quantifying redox-based changes within the plant proteome under oxidative stress conditions. A typical redox proteome labeling method uses either direct labelling of free reduced thiols or blocking labeling of disulfides/reversibly oxidized thiol groups with an alkylating agent such as *N*-ethylmaleimide (NEM) to block free cysteines, followed by DTT mediated reduction to reduce oxidized cysteines to Cys-SH and a subsequent labelling with fluorescent dye such as 5-iodoacetamidofluorescein (IAF) or monobromobimane (mBBr) [79]. Proteins are then separated by 2-DE and identified by LC-MS/MS technique. One advantage of using a dye like mBBr or IAF is a direct visualization of separated redox active proteins on the UV transilluminator. Moreover, shotgun proteomics approach has been exploited for identification of thiol-containing proteins selected as sub-proteomes trapped on activated thiol sepharose (ATS) beads [81]. Gel-free method exploiting derivatization of carbonylated proteins with 2,4-dinitrophenylhydrazine (DNPH) followed by tryptic digestion and enrichment by reversed phase chromatography coupled with MS/MS (RPC-MS/MS) or ion exchange and reversed phase chromatography coupled with MS/MS (IEC/RPC-MS/MS) has been successfully used for identification of carbonylated proteins and their oxidation sites [82]. 

Another major challenge for quantitative soybean proteomics is separation and identification of protein isoforms/species. During the course of evolution, soybean genome has undergone two rounds of whole genome duplication and many tandem duplication events [83]. Due to higher gene duplication and recombination process, so many protein isoforms exist in soybean as compared to rice and Arabidopsis. The 2-DE based proteomic techniques have a wide application in identifying these isoforms. Protein species occupy different positions on the 2-DE gel matrix based on their individual isoelectric point (p*I*) and relative molecular weight (MW), but share the same identification. Over the gel-based proteomic approach, bottom-up LC-MS/MS technique offers more advantages in identifying protein species. This method comprises of unambiguous identification of a single protein species relies on the identification of at least one peptide sequence that is uniquely found in that protein species [84]. Proper selection of the database would further facilitate the identification of such protein species with accuracy.

## 6. Conclusions

Instead of several limitations and challenges, soybean proteomics has proved itself as a valuable tool for identifying stress responsive target proteins with a clear picture of translational and post translational modification. More research works at the proteome level need to be undertaken for better understanding the minute changes in a cell’s protein signature to cope with the flooding and drought stress. Comparative organelle proteomes studies would be a great contribution towards understanding the cross-talk between stress signaling pathways. The convergence of diverse MS techniques coupled with bioinformatics technology with improved sample preparation and fractionation strategies is further needed to get a more precise and comprehensive picture of plant stress response mechanisms. 
